# Acute effects of two caffeine doses on bar velocity during the bench press exercise among women habituated to caffeine: a randomized, crossover, double-blind study involving control and placebo conditions

**DOI:** 10.1007/s00394-021-02708-8

**Published:** 2021-10-19

**Authors:** Aleksandra Filip-Stachnik, Michal Krzysztofik, Juan Del Coso, Michal Wilk

**Affiliations:** 1 Institute of Sport Sciences, The Jerzy Kukuczka Academy of Physical Education, ul. Mikolowska 72a, 40-065 Katowice, Poland; 2 Centre for Sport Studies, Rey Juan Carlos University, Fuenlabrada, Spain

**Keywords:** Caffeine tolerance, Ergogenic aids, Resistance exercise, Sports performance, Upper limbs

## Abstract

**Purpose:**

The main goal of this study was to evaluate the effectiveness of two different doses of caffeine (3 and 6 mg/kg) to enhance bar velocity during the bench press in women habituated to caffeine.

**Methods:**

Twelve recreationally trained women (age: 23.3 ± 0.8 years, body mass: 60.7 ± 5.7 kg, bench press one-repetition maximum (1RM): 44.3 ± 7.8 kg, daily caffeine ingestion: 5.7 ± 2.0 mg/kg/day) participated in a randomized double-blind experimental design. Each participant performed four different experimental sessions: after no supplementation (control, CON), after ingesting 3 and 6 mg/kg of caffeine (CAF-3 and CAF-6, respectively), or after ingesting a placebo (PLAC). In each experimental session, the participants performed 3 sets of 3 repetitions of the bench press exercise at 50% 1RM.

**Results:**

A two-way repeated-measures ANOVA with subsequent post hoc analyses indicated significant increases in peak velocity (*p* < 0.01; ES = 0.91) and mean velocity (*p* < 0.01; ES = 0.78) after the intake of CAF-6 compared to CON. The study did not show significant differences in bar velocity between CAF-6 and PLAC and between CAF-3 and PLAC. No significant differences in bar velocity were observed between CAF-3 and CAF-6 conditions.

**Conclusion:**

These results suggest that 6 mg/kg of caffeine can be an effective dose to improve power-specific training outcomes in women habituated to caffeine. However, the ergogenic effect of 6 mg/kg of caffeine may be derived from a combination of biological effects and expectancy, as this dose was only superior to the control condition with no differences over the placebo.

## Introduction

Caffeine is the most widely consumed psychoactive substance in the world [1] and one of the most comprehensively examined ergogenic substance for sports performance [2]. The underlying motivations of its ubiquitous use in modern society are supported by cognitive enhancement, as well as physical performance improvement [1]. As such, it is frequently used by both athletes and the general population alike. There are reports indicating that ~ 75% of high-performance athletes consume caffeine before or during competition [3], while there has been an increase in the amount of caffeine used in some athletes, such as weightlifters since caffeine was removed from the banned list of the World Antidoping Agency [4]. Additionally, recent evidence suggests that more than 85% of adults in the US consume caffeine on a regular basis, with an average daily intake of about 180 mg/day [5]. Collectively, these results suggest that a significant part of the population may be habituated to caffeine, particularly high-performance athletes.

It has been shown that habitual caffeine intake may dampen the physiological and cognitive effect of acute caffeine intake [6, 7]. The progressively lower effect of acute caffeine intake through habitual caffeine consumption may be explained by the up-regulation of adenosine receptors that occurs with a frequent exposition of caffeine [7], which would be responsible for attenuating caffeine’s stimulatory effects through the blockade of adenosine receptors [8]. However, studies analyzing the ergogenic effect of caffeine among participants who are habitual caffeine users are still inconclusive to determine if chronic consumption inhibits or reduces the ergogenic properties of acute caffeine intake. Several studies report no association between habitual caffeine intake and the magnitude of exercise performance improvement following acute caffeine ingestion in naïve vs habitual caffeine users [9–11]. On the contrary, some studies showed progressively diminished ergogenic effects of caffeine in individuals undergoing chronic ingestion of caffeine up to 28 days [12, 13]. Lastly, lower [14–17], or no effect of acute caffeine intake on exercise performance has been reported in habitual caffeine users [18], suggesting tolerance to caffeine when it is consumed chronically.

Although physiological changes have been found after chronic exposure to caffeine, which may be responsible for the suppressed ergogenic responses of acute caffeine intake in habitual caffeine users, a recent article by De Salles Painelli et al. [19] underlined the possible contribution of psychological aspects to this phenomenon. Previous experience in caffeine use and subsequent habituation has been indicated as factors that provide learning and information about the "new–old" treatment (i.e., acute caffeine intake) [19]. In this regard, it can be assumed that participants who consume caffeine regularly identify the caffeine-associated feelings after acute caffeine ingestion. Furthermore, caffeine may also promote side effects, such as increased alertness, heart rate, and blood pressure [1]. Consequently, as a result of self-observation, participants may correctly guess their allocation in the caffeine or placebo trial, which may influence performance assessment. Additionally, the belief in ingesting an active/inert substance may change the participants’ motivation leading to enhanced performance responses [20]. To avoid this problem, checking blinding efficacy and incorporating a baseline trial with no ingested substance (acting as a control situation) has been indicated as a key factor in caffeine research, particularly when designing experiments in individuals habituated to caffeine [19]. Unfortunately, only a few studies investigating acute caffeine effects on exercise performance reported data on the efficacy of blinding and used control conditions at the same time [19, 21] while none was carried out in habitual caffeine consumers.

Taking into account that women are still under-represented in caffeine research, especially in studies conducted on habitual caffeine users [22], the main goal of this study was to assess the acute effects of caffeine on bar velocity during the bench press exercise in women habituated to caffeine. Considering previously mentioned methodological concerns [19], we included four different experimental situations: a control trial with no treatment, a placebo trial and two caffeine conditions, with 3 and 6 mg of caffeine per kg of body mass. We selected these doses because recent evidence shows that an acute dose of caffeine similar to the daily caffeine intake is needed to obtain performance benefits of caffeine [23]. Based on previous studies [19, 23–25] and in the daily caffeine intake of the study sample, we hypothesized that acute caffeine ingestion only in the higher dose (*i.e.,* 6 mg/kg) would enhance performance compared to both control and placebo conditions.

## Materials and methods

### Experimental design

This study used a randomized, counterbalanced, double-blind, placebo-controlled crossover design where each participant acted as her own control. Participants performed a familiarization session with a pre-experimental bench press one-repetition maximum (1RM) measurement, followed by four experimental sessions. A minimum of 3 and a maximum of 7 days interval was provided between the sessions. On the first visit, participants performed a control session (CON) in which participants performed the exercise protocol without ingesting any substance. Then, the participants took part in three identical experimental sessions, in which they ingested either a placebo (PLAC), 3 mg/kg of caffeine (CAF-3) or 6 mg/kg of caffeine (CAF-6). The order in these three trials was assigned randomly. Both, caffeine and the placebo, were administered orally 60 min before the onset of the exercise protocol to allow peak blood caffeine concentration and at least 2 h after their last meal to avoid the influence of feeding on absorption rates. In the CON group, participants did not ingest any capsule, but they remained seated for 60 min to replicate the conditions of the remaining experimental conditions. Caffeine was provided in the form of commercially available capsules (Olimp Laboratories, Dębica, Poland). The manufacturer of the caffeine capsules also prepared identical PLAC capsules filled with an inert substance (all-purpose flour). The blinding and randomization of the three experimental sessions with ingestion of a capsule were conducted by a member of the research team that was not directly involved in data collection. During all four experimental sessions, the participants performed 3 sets of 3 repetitions of the bench press exercise with a load equivalent to 50%1RM, as measured in the pre-experimental trial. At the end of each session, the efficiency of blinding and occurrence of side effect during the trial was checked. The trials were performed at the same time of the day (in the morning, between 9:00 and 12:00) and in a laboratory room with controlled ambient temperature (~ 21 °C). The study protocol was approved by the by the University Ethics Committee in accordance with the latest version of the Declaration of Helsinki.

### Study participants

To calculate the sample size, the statistical software G*Power (Dusseldorf, Germany) was used with the following variables: ANOVA, repeated-measures, within factors was assumed as the statistical test, small expected effect size (ES) for caffeine on bar velocity = 0.21 (obtained from recently performed meta-analysis analyzing movement velocity in resistance exercise [26]), alpha = 0.05, the statistical power = 80%, *r* = 0.85, one group of participants, and four experimental conditions. The power analysis indicated that a sample of at least 11 participants was required to determine statistically significant differences with caffeine vs placebo condition on bar velocity. Based on the power analysis, and to avoid possible dropouts, we recruited 12 healthy recreationally trained women into the study. The main characteristics of the participants of the study are depicted in Table 1. The inclusion criteria were as follows: (a) free from neuromuscular and musculoskeletal disorders; (b) habitual caffeine user within the range of moderate-to-high consumption as per a previously proposed classification [22] (c) resistance-trained (defined as a minimum 2 years of resistance training experience with at least 3 days per week for the 6-month period prior to enrollment in this study) (d) not using any medications, dietary supplements or ergogenic aids which could potentially affect the study outcomes (e.g., beta-alanine, creatine) and (e) a self-described satisfactory health status as per an ad hoc questionnaire. Participants were excluded if they reported (a) a positive smoking status or (b) a potential allergy to caffeine.

**Table 1:** Participants’ characteristics

Variable [units]	Value (mean ± SD)
Age [years]	23.3 ± 0.8
Body mass [kg]	60.7 ± 5.7
Height [cm]	166.6 ± 4.8
Body Fat [%]	20.0 ± 2.7
Resistance training experience [years]	2.9 ± 0.8
Bench press exercise 1RM [kg]	44.3 ± 7.8
Habitual caffeine intake [mg/kg/day; mg/day]	5.7 ± 2.0; 348.6 ± 135.6
Energy intake [kcal]	1957.8 ± 220.8
Protein [% of total energy intake]	18.2 ± 4.8
Carbohydrates [% of total energy intake]	54.8 ± 6.7
Fat [% of total energy intake]	27.1 ± 6.3

All data are presented as mean ± standard deviation

*1RM* one-repetition maximum

### Pre experimental standardization

Before the first experimental trial, participants were instructed to maintain their usual hydration and dietary habits and habitual caffeine intake and sleep patterns during the study period. In addition, the participants registered their food and fluid intake using “MyFitnessPal” software for 24 h before each experimental trial. To produce a within-subject standardization of diet, participants replicated the same dietary pattern before each trial. Habitual caffeine intake was measured using a modified version of the validated questionnaire by Bühler et al. [27] that recorded the type and amount of caffeine-containing foods and dietary supplements. Habitual caffeine intake was assessed for the four weeks before the start of the experiment, following previous recommendations [22]. Six participants were classified as moderate, while the other six were classified as high caffeine users [22]. Participants were also asked to refrain from any source of caffeine for 12 h before each experimental trial and not to perform strenuous exercise for the 24 h before testing. Adherence to these requirements was verified via a brief questionnaire administered before data collection in each trial.

### One repetition maximum test and familiarization session

One week before the onset of the experimental trials, the participants underwent a 1RM test followed by a familiarization with the study protocol. On this day, the participants arrived at the laboratory at the same time of the day as in the upcoming experimental sessions. First, the following anthropometric measurements were taken: height (WPT-60/150OW, Radwag, Poland), body mass and body fat percentage (InBody 370, Biospace Co., South Korea). Then, the participants performed their habitual warm-up protocol for 15 min that included upper body and lower body resistance exercise with increasing loads and at increasing velocity. Then, the participants performed a one-repetition maximum test in the bench press exercise described elsewhere [24]. Hand placement on the barbell was individually selected with a grip width on the barbell of 150% individual biacromial distance. After completing the 1RM test, the participants performed the familiarization session which consisted of 3 sets of 3 repetitions with 50% of the 1RM, as evaluated previously.

### Experimental protocol

The four experimental sessions were identical, except for the lack of capsule ingestion in the control trial. All testing took place between 9.00 and 12.00 am to avoid the effect of circadian variation on the study outcomes. After their habitual warm-up, and 60 min after the ingestion of the substances, the participants began the exercise consisting of 3 sets of 3 repetitions at 50%1RM in the bench press exercise. The rest interval between sets equaled 3 min. The eccentric and concentric phases of the bench press exercise were performed at their maximal possible velocity in each repetition. Execution technique and motivation were standardized and monitored by two experienced researchers. A linear position transducer system (Tendo Power Analyzer, Tendo Sport Machines, Trencin, Slovakia) was used for the measurement of bar velocity. During each repetition, peak bar velocity and mean bar velocity (m/s) were registered. Mean velocity was obtained as the mean of the three repetitions, while peak velocity was obtained from the best repetition in each set. At the end of each session with the ingested substance, the participants were asked to guess which substance they had ingested (placebo vs caffeine) and the dose of caffeine (3 vs 6 mg/kg) to determine the efficacy of blinding procedures.

### Side effects

Immediately after finishing testing, and after 24 h of ingesting the substances, participants were asked about typical caffeine-associated side effects using a questionnaire that obtained the prevalence of these side effects with dichotomic yes/no responses [16, 28]. Additionally, the participants were asked about increased vigor/activeness perception of performance improvement during the testing. The questionnaire about side effects was not filled in the CON session.

### Statistical analysis

#### Performance

Data are presented as the mean ± SD. All variables presented a normal distribution according to the Shapiro–Wilk test. Verification of differences in peak bar velocity (peak velocity), and mean bar velocity (mean velocity) among experimental trials was performed using a two-way (4 conditions × 3 set) analysis of variance (ANOVA) with repeated-measures. The significance level was set at *p* < 0.05. In the event of a significant main effect, post hoc comparisons were conducted using the Tukey’s test. A one-way ANOVA was used to determine differences in energy intake and in the amount of protein, carbohydrate and fat in the participants’ diet for the 24 h before the trials. Effect sizes (Cohen’s *d*) were reported where appropriate and interpreted as large (*d* ≥ 0.80); moderate (*d* between 0.79 and 0.50); small (*d* between 0.49 and 0.20); and trivial (*d* < 0.20); [29].

#### Side effects

A Fisher’s Exact test in a contingency table was conducted to evaluate whether the caffeine dose was associated with the occurrence of side effects immediately and 24 h post exercise. The two variables included the dose with three levels (placebo, CAF-3, CAF-6) and the occurrence of side effects with two levels (yes and no). The significance level was set at *p* < 0.05. Moreover, a Cochran’s Q test with pairwise comparison was conducted to evaluate differences between doses in the occurrence of side effects. The magnitude of association between caffeine dose and the occurrence of side effects was described by Cramer’s *V* with the following approach: low (between 0.1 and 0.3), moderate (between 0.3 and 0.5) and high (> 0.5).

## Results

### Pre-trial standardization

The one-way repeated-measures ANOVA indicated no significant differences in energy intake (*p* = 0.93) and in the proportion of protein (*p* = 0.37), carbohydrate (*p* = 0.06) and fat (*p* = 0.18) between conditions.

### Performance

The two-way repeated-measures ANOVA indicated no significant substance × set main interaction effect for peak velocity (*F*_3,11_ = 0.157; *p* = 0.99) and mean velocity (*F*_3,11_ = 1.35; *p* = 0.25). However, there was a significant main effect of substance for peak velocity (*F*_3,11_ = 6.60; *p* < 0.01) and for mean velocity (*F*_3,11_ = 5.48; *p* < 0.01; Table 2). Post hoc analyses for main effect of substance indicated significant increases in peak velocity (*p* < 0.01; ES = 0.91) and mean velocity (*p* < 0.01; ES = 0.78) after the intake of CAF-6 compared to CON condition. The analysis did not show significant differences in mean and peak bar velocity between CAF-6 and PLAC; between CAF-3 and PLAC; between CAF-3 and CAF-6; and between CAF-3 and CON condition. However, an effect of small magnitude was found when comparing PLAC and CON while this effect was of moderate size when comparing CAF-3 and CON condition. The results of mean velocity and peak velocity in individual sets for CON, PLAC, CAF-3 and CAF-6 conditions are presented in Table 3.

**Table 2:** Average values and effect size of mean and peak bar velocity during 3 sets of the bench press exercise at 50% 1RM with the ingestion of 3 and 6 mg/kg of caffeine, with a placebo or in a control situation without substance ingestion in resistance-trained women habituated to caffeine

Average values					
	CON	PLAC	CAF-3	CAF-6	Main effect of substance
Mean bar velocity [m/s]	0.86 ± 0.08	0.90 ± 0.10	0.91 ± 0.10	0.94 ± 0.12	< 0.01
Peak bar velocity [m/s]	1.18 ± 0.11	1.24 ± 0.13	1.25 ± 0.16	1.31 ± 0.17	< 0.01

Effect size [Cohen’s *d* units]						
	CON vs CAF-6	PLAC vs CAF-6	CON vs PLAC	CON vs CAF-3	PLAC vs CAF-3	CAF-3 vs CAF-6
Mean bar velocity	0.78	0.36	0.44	0.55	0.10	0.27
Peak bar velocity	0.91	0.46	0.5	0.51	0.07	0.36

Average values of mean and peak bar data are presented as mean ± standard deviation

*CON* control, *PLAC* placebo, *CAF-3* caffeine 3 mg/kg, *CAF-6* caffeine 6 mg/kg

**Table 3:** Mean and peak bar velocity for each one of the 3 sets of the bench press exercise performed at 50% 1RM with the ingestion of 3 and 6 mg/kg of caffeine, with a placebo or in a control situation without substance ingestion in resistance-trained women habituated to caffeine

Conditions	Set 1	Set 2	Set 3
Mean bar Velocity [m/s]			
CON	0.86 ± 0.10	0.87 ± 0.09	0.85 ± 0.06
PLAC	0.89 ± 0.10	0.89 ± 0.12	0.90 ± 0.08
CAF-3	0.93 ± 0.08	0.90 ± 0.13	0.90 ± 0.11
CAF-6	0.93 ± 0.13	0.97 ± 0.11	0.91 ± 0.12
Peak Bar Velocity [m/s]			
CON	1.18 ± 0.13	1.20 ± 0.14	1.17 ± 0.09
PLAC	1.24 ± 0.13	1.25 ± 0.16	1.22 ± 0.12
CAF-3	1.24 ± 0.17	1.27 ± 0.18	1.25 ± 0.15
CAF-6	1.30 ± 0.20	1.32 ± 0.17	1.29 ± 0.17

All data are presented as mean ± standard deviation

*CON* control, *PLAC* placebo, *CAF-3* caffeine 3 mg/kg, *CAF-6* caffeine 6 mg/kg

### Side effects

A Fisher’s Exact Test showed a statistically significant and moderate association between caffeine dose and perception of performance improvement (*p* = 0.045; Cramer’s *V* = 0.416) and increased vigor/activeness (*p* = 0.02; Cramer’s *V* = 0.471) immediately post exercise, with no statistically significant associations in the remaining effects immediately and 24 h post exercise (Table 4). The Cochran’s *Q* tests revealed a statistically significant difference for increased vigor/activeness (*p* = 0.034) immediately post experiment. The pairwise comparisons indicated a statistically significant greater increased vigor/activeness for CAF-6 versus PLAC (*p* = 0.028). No other statistically significant differences in the prevalence of side effects between caffeine doses or after caffeine intake respect to PLAC and CON were found.

**Table 4:** Frequency of side effects immediately after and 24 h after a bench press exercise session with the ingestion of 3 and 6 mg/kg/ of caffeine with a placebo or in a control situation without substance ingestion in resistance-trained women habituated to caffeine.

	PLAC		CAF-3		CAF-6	
	Just after	24 h after	Just after	24 h after	Just after	24 h after
Muscle soreness (%)	0	8	0	0	8	0
Increased urine output (%)	8	17	17	8	0	8
Tachycardia and heart palpitations (%)	0	0	25	8	25	8
Anxiety or nervousness (%)	17	8	17	8	33	8
Headache (%)	17	33	8	8	8	17
Gastrointestinal problems (%)	0	0	8	0	8	8
Insomnia (%)	–	0	–	8	–	8
Increased vigor/activeness (%)	0	8	25	0	50*	0
Perception of performance improvement (%)	8	–	42	–	33	–

Data are presented as the frequency that responded affirmatively to the existence of a side effect

*PLAC* placebo, *CAF-3* caffeine 3 mg/kg, *CAF-6* caffeine 6 mg/kg

*Significant difference (*p* < 0.05) between CAF6 and PLAC

### Assessment of blinding

Out of the twelve participants in the investigation, five (42%) correctly identified PLAC and CAF-3. Eight participants (67%) correctly identified CAF-6 conditions. Additionally, eight (67%) and eleven (92%) of the 12 participants indicated that they had ingested caffeine in CAF-3 and CAF-6 trials, respectively.

## Discussion

The main finding of this study was that only the acute intake of 6 mg/kg of caffeine had a statistically significant performance benefit on bar velocity during the bench press exercise consisting of 3 sets of 3 repetitions at 50% 1RM in women habituated to caffeine. Specifically, an increase in performance was observed in mean and peak velocity after the intake 6 mg/kg of caffeine compared to the CON trial where there was no capsule ingestion. However, the study did not show statistically significant differences in bar velocity between placebo and the ingestion of 3 or 6 mg/kg of caffeine. These results suggest that ingesting 6 mg/kg of caffeine are necessary to obtain an improvement in power-specific training outcomes in women with moderate to high levels of caffeine consumption. The lack of differences between caffeine and placebo trials, and the high rate of identification of caffeine trials, suggest that the ergogenic benefit derived from 6 mg/kg of caffeine was associated to pharmacological effects derived from substance intake plus expectancy. The absence of ergogenic aid with caffeine intake over the placebo, despite the same doses has been confirmed as ergogenic in previous investigations in respect to placebos [21, 28, 30–32], is probably linked to the habituation to caffeine in the study sample, which ingested 5.7 ± 2.0 mg/kg/day of caffeine daily. It is likely that habituation to caffeine produced tolerance to the ergogenic effect of caffeine [13] as both doses were below their daily caffeine intake. Collectively, a protocol of supplementation with 6 mg/kg of caffeine is needed to obtain partial ergogenic benefits in moderate female consumers of caffeine [22] when performing resistance-based exercise, while expectancy may have a definitive role in obtaining caffeine’s ergogenic effect in this population. Second, this study indicates that collecting baseline values during a control condition with no substance ingestion might be necessary for proper assessment of the impact of caffeine in habituated participants, as the impact of the placebo effect may be diminished by the identification of ingested substance. In this regard, the pharmacological effect of caffeine and subject-expectancy effect are obtained together when supplementing with caffeine in real sports scenarios.

Previous research has shown that the acute ingestion of a moderate dose of caffeine (from 3 to 9 mg/kg of caffeine) has the potential of enhancing several aspects of strength-power performance of upper-limbs [10, 11, 15, 21, 28, 33]. However, most of the aforementioned investigations have been carried out in participants with low daily caffeine intake. As previously found in other forms of resistance exercise, the ingestion of a moderate dose of caffeine may not be ergogenic, or at least less ergogenic, in individuals habituated to caffeine [23] or the use of high doses of caffeine (i.e., ≥ 9 mg/kg) may be needed to obtain such performance benefits in resistance exercise performance [16, 17]. The results of the present study are in line with previous investigations because they suggest that women habituated to caffeine through a moderate level of daily consumption only obtained enhanced bar velocity performance during the bench press exercise with 6 mg/kg of caffeine, while such changes were not observed after the ingestion of 3 mg/kg of caffeine. In fact, Pickering and Kiely [34] suggested that habitual caffeine users may maintain the ergogenic effect of acute caffeine intake using doses higher than the mean daily level of caffeine consumption. In the current investigation, this theory was partially supported because only the dose that was similar to their daily caffeine intake was effective to enhance performance. It is worth noting that the improvement in bar velocity after the ingestion of 6 mg/kg of caffeine was observed only in comparison to the CON condition, while the differences were not statistically significant when comparing CAF-6 and PLAC. Given that habituation to caffeine through daily intake has been suggested as a factor that provides learning and information about the responses to acute caffeine intake [25], the use of a control condition in the current study may have been particularly important to detect the ergogenic effect of 6 mg/kg of caffeine in a group of women habituated to caffeine. This is because individuals habituated to caffeine may benefit more from expectancy and they are more prone to identify the substances ingested. Furthermore, results of the studies using psychostimulant substances [35, 36], suggested that women have a greater propensity to produce placebo responses of a higher magnitude and duration than men. Thus, the lack of differences between PLAC and CAF-6 condition may be explained by the fact, that the PLAC trial did not act as a neutral, “control” condition in the sample of women habituated to caffeine. Although there were no significant differences between CON and PLAC conditions in bar velocity, the magnitude of the effect of PLAC over the CON was from small to moderate (Table 2), which suggests an improvement due to psychological reasons under PLAC conditions. Collectively, this information suggests that in women habituated to caffeine, the use of a dose equivalent to their daily caffeine intake may be needed to obtain performance benefits on power-based resistance exercise performance.

In addition to habituation to caffeine, other factors may be associated with the lack of ergogenic effects of caffeine, in a dose of 3 mg/kg, on performance. For example, previous studies that observed an acute positive effect after the intake of 3 mg/kg [10, 11, 15, 21, 28, 33] were conducted on men or a mixed-gender population. On the contrary, the present study only considered of women, what can explain the difference between the obtained results. The results from a recently performed systematic review [37] indicated that there are some subtle differences between sexes resulting in the increased ergogenic effect of caffeine on resistance exercise performance in men than in women, despite similar dosage and training level. This phenomenon might be supported by the fact that the increase in neuromuscular activity that facilitates neural transmission is observed in men, and there is no such evidence in women [38]. Additionally, the study of Adan et al. [39] has shown that some of the stimulant effects after the intake of low caffeine doses are greater in men compared to women. Taken together, the use of women as a study sample could also explain the differences between responses observed in previous studies conducted on male subjects [10, 11, 15, 21, 28, 33], and results of this study with women participants.

Additionally, in the presented study, 67% of participants correctly identified the trial with 6 mg/kg of caffeine and 92% of participants indicated the use of caffeine in either of the trials with caffeine intake. De Salles Painelli et al. [19] indicated that correct guessing of the allocation in caffeine trials may positively regulate exercise performance. Confirmation of that theory is observed in the presented research, since after the intake of CAF-6, 50% of participants reported increased vigor and activeness during the experiment, which also may impact participants’ motivation and self-efficacy, leading to enhanced performance responses [20]. Interestingly, previous research showed that the synergistic effect of the pharmacological and psychological influence of nutritional interventions lead to the greatest improvements in sport, exercise and cognitive performance [40–42]. Thus, it is possible that positive effects of CAF-6 in the presented research have occurred due to the synergistic impact of pharmacological and psychological influence of caffeine. In general, the results of the presented research support the notion that the psychological permutations associated with caffeine habituation may significantly influence caffeine ergogenic aids.

In addition to its strengths, the present study has several limitations which should be addressed: (1) the study did not include any biochemical analysis, such as plasma/urinary caffeine concentrations, which could help explain the obtained results. However, the dose–response effect of caffeine (Table 2) together with the high percentage of individuals that identified when caffeine was administered suggest that the outcome of this investigation was the result of the treatments used; (2) only two doses of caffeine were investigated while it is still possible that doses higher than 6 mg/kg of caffeine may produce greater performance benefits in a sample of women habituated to caffeine (3) the study sample only included women with moderate-to-high caffeine consumption, and the generalizability of these results to men or women with other levels of caffeine consumption is questionable (3); the hormonal changes as a result of the menstrual cycle were not controlled in the investigation. In any case, previous investigation suggests that the response to caffeine intake in resistance exercise [43] is similar across the menstrual cycle (4); the study sample was composed of individuals with a moderate level of strength, thus the translation of the research outcomes to highly trained women in resistance-based sports should be made with caution; (5) bar velocity was only measured during the concentric phase of the bench press exercise. Despite these limitations, we consider that the present investigation contributes to determine the ergogenic effects of caffeine on resistance exercise performance in women habituated to caffeine.

## Conclusion

The results of the current investigation showed an ergogenic effect of 6 mg/kg of caffeine on bar velocity during a bench press exercise routine consisting of 3 sets of 3 repetitions with a load equivalent to 50% 1RM in women with a daily caffeine intake of 5.7 ± 2.0 mg/kg. Interestingly, this effect was present in the control condition with no substance ingestion but not in the placebo condition. The high rating of identification of the order of the trials suggests that, beyond dosage, expectancy may have a definitive role for obtaining of caffeine’s ergogenic effect in this population. The effect of 3 mg/kg of caffeine did not reach statistical significance likely because this dose was below the daily caffeine intake of the participants. From a practical perspective, in women athletes habituated to caffeine, ingesting an acute a dose of caffeine equivalent to the amount of caffeine ingested daily may be needed to obtain the ergogenic benefits of this stimulant on bench press performance. The magnitude of the ergogenic effect of 6 m/kg of caffeine was large in respect to the control condition, suggesting that caffeine supplementation is a potent substance to enhance muscular performance during resistance-based training or during competition. In case of choosing caffeine supplementation as an ergogenic aid, the minimal effective dose should be considered and their potential ergogenic effect balanced over its drawbacks as some of caffeine’s side effects increase along chronic intake.

## Declarations

### Availability of data and material

The datasets used and/or analyzed during the current study are available from the corresponding author on reasonable request.
